# Hemangioma of penile urethra—treatment with simple transurethral excision: a case report

**DOI:** 10.4076/1757-1626-2-6199

**Published:** 2009-07-14

**Authors:** Ioannis Efthimiou, Diamantis Kavouras, Panagiotis Vasilakis, Spiridon Katsanis

**Affiliations:** 1 Department of Urology, General Hospital of Chios “Skilitseio”Chios, TK 82100Greece; 2 Department of Urology, University Hospital of PatrasPatraGreece; 3 Department of Urology, General Hospital of “Agia Olga”AthensGreece

## Abstract

Urethral hemangiomas are rare benign vascular tumors with varying size and usually present as urethral bleeding and/ or hematuria. Treatment depends on the size and site of the lesion. We present a 27 year old male with a two year history of intermittent episodes of urethral bleeding. Cystourethroscopy showed a solitary hemangioma in the penile urethra. The patient was treated with simple transurethral excision with the biopsy forceps. The catheter was removed 48 hours later. He remains symptom free four months later. Simple excision of small hemangiomas may be an effective treatment especially for young patients in order to avoid the side effects of diathermy and when facilities such is laser are not available.

## Introduction

Urethral hemangiomas are rare benign vascular tumors which on histology consist of thin walled vascular spaces lined by endothelial cells. Their origin still remains an enigma. It has been suggested they originate from unipotent angioblastic cells that fail to develop into normal blood vessels. The most common type is cavernous hemangioma [1]. Treatment may be extremely challenging and ranges from transurethral approach to open reconstructive surgery. Herein we present a case of a young male who was treated with simple transurethral excision of the lesion with the biopsy forceps.

## Case presentation

**Figure 1.**
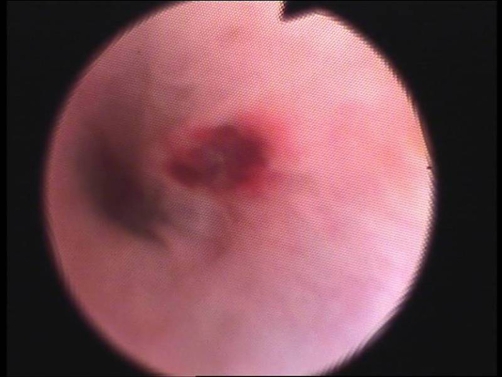
Endoscopic view of the hemangioma of the penile urethra in the patient.

**Figure 2.**
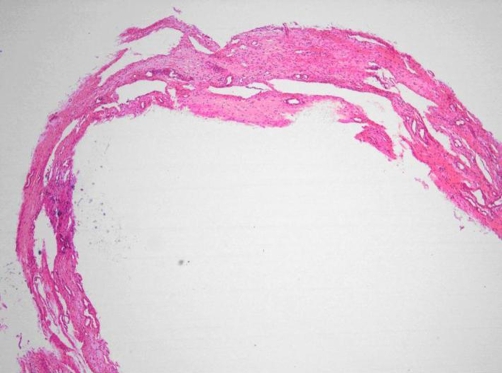
Light microscopic photograph of the cavernous hemangioma. There is accumulation of subepithelial thin vessels (H & E x 20).

A 27 year old white man presented in our hospital with a two years history of intermittent urethral bleeding. He did not describe any bladder symptoms or any kind of urethral trauma. All the episodes were self-limited. Many of them had been treated as recurrent episodes of urethritis. The patient reported episodes of urethral bleeding and initial hematuria 2-3 times per week and sometimes 2-3 times per day, without any obvious precipitating factor. He did not report any hemospermia. On physical examination vital sings were normal. Examination of the abdomen and external genitalia was normal. The urethral meatus had a normal caliber. There was urethral discharge of fresh blood. The prostate gland was soft, small and non tender on digital rectal examination. Midstream urine analysis was normal and urethral culture of swabs for *N. Gonorrhoea* was negative. Biochemical, haematological and clotting studies were normal. Ultrasound scan of the kidneys and bladder was normal. The urethral bleeding was controlled with an indwelling catheter 16 Fr which was kept in place for 48 hours. Flexible cystoscopy under local anaesthesia showed a reddish lesion 5 mm about 5 cm from the external urethral meatus (Figure 1). The patient was scheduled for transurethral excision and biopsy of the lesion. Under general anaesthesia and with the use of the 5 Fr flexible biopsy forceps the lesion was excised. A 20 Fr urethral catheter was placed for 48 hours. Histological examination of the specimen revealed a urethral cavernous hemangioma (Figure 2). Four months after treatment the patient continues to be symptom-free without any episode of urethral bleeding.

## Discussion

Urethral hemangiomas can occur at any age. Most cases are encountered in males and only a few have been reported in female [2]. They are often associated with the presence of cutaneous hemangiomas and have also been associated with Klippel-Trenaunay syndrome [3]. Presentation depends on the site and size of the lesion. Hemangiomas of the anterior urethra may present as urethral bleeding and lesions located in the proximal urethra usually present as hematuria, urinary retention with blood clots, post-erection or post-ejaculation hematuria and hemospermia [1]. Large lesions may present with obstructive urinary symptoms or protrude through the urethral meatus [1,4]. Urethroscopy is the diagnostic method of choice as it defines the site and extent of the lesions and facilitates the preoperative plan of surgery. Posterior urethra hemangiomas typically present between the veromontanum and external sphincter. Ultrasound examination of the penis or transrectal power Doppler ultrasound may also be helpful as they may show thickening of the soft tissues and a strong blood flow respectively [1,5]. Larger lesions, especially those protruding from the meatus have more diagnostic challenges as they must be differentiated from warts, polyps, malignant tumors, abscess and caruncle.

Asymptomatic lesions do not require any treatment but extensive lesions may require open excision and urethral reconstruction [6]. Treatment with laser may obviate the need for open and extensive surgery. Hemangiomas treated with KTP, Nd: YAG and holmium laser all reported excellent results [5,7,8]. Also selective arterial embolisation has been reported [9]. Elecrofulguration has been used in the past but carries the risk of scarring [10]. In our case, as our hospital does not have the facility of laser technology, we selected to treat the patient with simple transurethral excision of the lesion with the biopsy forceps without the use of electrofulguration. The patient after four months still remains symptom free. Although cystoscopic excision is much less invasive, it has not been well described in literature and we should keep in mind that diathermy or other more invasive approaches such is selective arterial embolization may be needed to control bleeding from the feeding vessel because placing a catheter may not always control the bleed. Careful preoperative planning is required in order to determine the depth of the lesion as what we see cystoscopically may just be the tip of the iceberg.

## Conclusions

Simple transurethral excision of small urethral hemangiomas may be an effective treatment especially for young patients in order to avoid the side effects of diathermy and when laser facilities are not available. The cornerstone of the right management of urethral hemangiomas is still careful preoperative definition of the extension of the lesion.

## Abbreviations

KTP: potassium titanyl phosphate
Nd:YAG: neodymium:yttrium-aluminum-garnet laser
